# Hippuric Acid as a Significant Regulator of Supersaturation in Calcium Oxalate Lithiasis: The Physiological Evidence

**DOI:** 10.1155/2013/374950

**Published:** 2013-11-07

**Authors:** Stoyanka S. Atanassova, Ivan S. Gutzow

**Affiliations:** ^1^Department of Clinical Laboratory and Clinical Immunology, Medical University, “G. Sofiisky” Boulevard 1, 1431 Sofia, Bulgaria; ^2^Institute of Physical Chemistry, Bulgarian Academy of Sciences, 1113 Sofia, Bulgaria

## Abstract

At present, the clinical significance of existing physicochemical and biological evidence and especially the results we have obtained from our previous *in vitro* experiments have been analyzed, and we have come to the conclusion that hippuric acid (C_6_H_5_CONHCH_2_COOH) is a very active solvent of Calcium Oxalate (CaOX) in physiological solutions. Two types of experiments have been discussed: clinical laboratory analysis on the urine excretion of hippuric acid (HA) in patients with CaOX lithiasis and detailed measurements of the kinetics of the dissolution of CaOX calculi in artificial urine, containing various concentrations of HA. It turns out that the most probable value of the HA concentration in the control group is approximately ten times higher than the corresponding value in the group of the stone-formers. Our *in vitro* analytical measurements demonstrate even a possibility to dissolve CaOX stones in human urine, in which increased concentration of HA have been established. A conclusion can be that drowning out HA is a significant regulator of CaOX supersaturation and thus a regulation of CaOX stone formation in human urine. Discussions have arisen to use increased concentration of HA in urine both as a solubilizator of CaOX stones in the urinary tract and on the purpose of a prolonged metaphylactic treatment.

## 1. Introduction

Calcium oxalate stones are the most common type of stones in urolithiasis in developed countries [1–3]. In addition, it is very important that the formation of such stones is caused by some gastrointestinal diseases [4]. 

 Two main factors characterize a calcium oxalate (CaOX) calculus as the most important stone formed in our lives: on the one hand, this fact is true, because of its frequency of occurrence and a high rate of recurrence, but on the other hand, it is due to the lack of a significant therapeutic effect of a dietary or a pharmacological attack. Despite a number of promising hypotheses, the pathogenic mechanism of intrarenal calcium oxalate stone formation remains, in many aspects, obscure. We must also say that, based on the dominant role of hypercalciuria in the pathogenesis of calcium oxalate stone formation [5, 6], the therapeutic efforts that have been made, prove neither clinically nor scientifically to be well applied.

 The problem of calculating the supersaturation in urine (i.e., the driving force of this particular case of phase transformation) and the problem of finding out the possible natural or pharmaceutical regulators of this driving force, in the process of investigating the kinetics of crystal nucleation and growth, as well as in the investigation of the kinetics of the dissolution of already existing stones, appears to be of utmost significance. 

Although the relative effect of a number of constituents of human urine on the solubility of CaOX has been determined in simple salt solutions, the exact combination of the factors that are responsible for variations in CaOX solubility in urine is very yet insufficiently known. Bearing this in mind, changes in the urinary oxalate ion concentration are more likely to decrease the supersaturation of urine than similar changes in the urine calcium concentration. Moreover, we know that the amount of oxalate excreted in urine depends on endogenous production, intestinal absorption, dietary intake, and renal transport. The endogenous production of oxalate, predominantly derived from the metabolism of glyoxylate and ascorbates, contributes significantly to the amount of oxalate that is excreted in urine [7–9]. We also know that it is possible to influence oxalate endogenous production by drug administration. In this connection, a number of clinical studies have demonstrated that the intake of certain drugs, which are essentially benzoate derivatives, leads to an increase in CaOX solubility [10, 11]. The main route of biotransformation of benzoic acid in human individuals is conjugation with glycine, which results in the formation of hippuric acid (HA) [12–14].

 In previous researches (see [15, 16]), we have found out that one of the normal physiological constituents of human urine—hippuric acid (C_6_H_5_CONHCH_2_COOH)—is, in fact, a complex forming physiological solvent of calcium oxalate. Our *in vitro *experiments showed that in the presence of HA, the solubility of CaOX increases considerably and that in physiological solutions, in which HA is in concentrations approximately five to six times over its normal physiological concentration in urine (5 to 14 mMol/24 h, according to the data presented by Boshev as given in [17]), the supersaturation in the system decreases significantly. These results, an account of which is given in [15, 16], are most meaningful from a physiological point of view, considering the well known fact that the increase of the concentration of HA in urine can be provoked by the *per os *administration of benzoic acid derivatives. This circumstance is, in fact, used in Quick's well known test of liver function [14, 18]. This test reflects on the metabolism of aromatic complexes in the human organism and it is a demonstration of the long ago established fact that HA is the end product of the detoxification of aromatic complexes in human individuals. 

 It has been proven in recent years that the supersaturation in urine is determined not so much by the concentration of ions, constituting the concrements, but rather more by the presence or absence of complex forming ions in urine. This has been evident since Hammarsten's classic studies [19], according to which many ions, such as Mg^2+^, or citrate ions, which are also normally present in urine, increase the solubility of CaOX in aqueous solutions by forming complexes with either the Ca^2+^ or the C_2_O_4_^2−^ ions. Thus, Mg^2+^ and citric ions are to be considered as the two regulators of supersaturation in urine, concerning CaOX precipitation. However, the oral administration of these two complexing agents does not lead to encouraging clinical results, as they undergo metabolization in the human organism. From our already quoted investigations [15, 16], it follows that the concentration of HA also determines, essentially, the solubility of CaOX and that this substance may be the third and even most significant biological regulator of CaOX supersaturation in human urine. 

 Up to now, no biological or biochemical analysis has been performed concerning the possible correlation between HA concentration in urine and the inclination to calcium oxalate calculosis. The experimental investigations mentioned above and obtained in our *in vitro* experiments on the dissolution of CaOX in physiological solutions, containing an increased concentration of HA, necessitated such a generalized physiological analysis and the results of which are given below.

## 2. Patients and Methods

Two types of experiments were performed in the framework of our investigation.

(1) Clinical laboratory analysis on the concentration of HA in urine and the amount of total urine excretion of HA in patients with CaOX stones, as well as, in a healthy control group; and (2) an investigation on the kinetics of the dissolution of CaOX calculi in physiological solutions, containing various concentrations of HA.

### 2.1. Patients Studied

#### 2.1.1. Stone Formers (SFs)

56 patients (30 men and 26 women), whose age ranged from 14 to 65 years, at the beginning of the disease, and who had had their calcium oxalate renal calculi removed (spontaneously, by surgery or through extracorporeal lithotripsy (ESWL)). Each patient had a known clinical history of his disease, including data for episodes of a renal colic and of concrements elimination, real urolithiasis in their families, the presence of metabolic disorders, and so forth. During the conduction of the study, the patients were on free mode and diet and with a fully compensated renal function. They did not report any liver disease. All investigated patients were hospitalized in the Clinic of the Department of Urology at the Medical University, Sofia.

#### 2.1.2. Controls

15 healthy subjects who had never had any urological and hepatic trouble.

#### 2.1.3. Methods

A 24-hour urine collection was obtained from each patient. During the period of urine collection, specimens were refrigerated and aliquots of the 24-hour volume sample were immediately frozen until analyzed. The volume of urine in every sample was recorded on completion of the collection and pH was measured by using a glass electrode pH-meter. 

The calcium, oxalate, and other substances, such as creatinine, magnesium, phosphorous, uric acid, were also determined. All citied substances were measured by automated known spectrophotometry and colorimetric analysis. The urine levels of oxalates were evaluated by enzymatic method [20].

Amino acid contents (hippuric acid, glycine, serine, and so forth) of the collected samples were determined using a Hewlett Packard HPLC 1050 instrument (high-performance liquid chromatography), coupled to a fluorescence detector. Ethyl alcohol was added to urine specimens to allow the precipitation of proteins and the extraction of free amino acids. An automated precolumn orthophtaldehyde derivation procedure was employed. Separations were done using a reversed-phase column (Waters Corp). Amino acid concentration of the samples was determined in comparison to the values obtained from a standard curve prepared for each amino acid. 

### 2.2. Statistical Analysis

Statistical analysis of the data obtained from both the SF patients and from the control group was performed using Student *t*-test, to establish the significance of the difference between mean values. All results were expressed as a mean ± SEM and differences were considered significant if *P* < 0.05.

### 2.3. Dissolution of CaOX Concrements with Hippuric Acid (HA): *In Vitro* Experiments

#### 2.3.1. Instrumental Techniques

The experiments on the kinetics of the dissolution of CaOX renal calculi were performed in Jena glass round bottom flasks thermostated at 25°C. The volume of the studied solution was 1000 mL and it was stirred (~200 rpm) by an electromagnetic stirrer. The Archimedean weight *G(t)* of the samples of CaOX calculi, put in a platinum net basket and suspended to a torsion balance, was continuously measured with a sensitivity of ±0.5 mg (see [15, 16]).

The CaOX calculi used had been formed in the urinary tract and eliminated spontaneously by the patients. The calculi were selected to have a weight of 100 to 200 mg and to be of identical mineral composition, mainly CaC2O4·2H2O (weddellite). The composition of the calculi was checked by polarized light microscopy and thermogravimetry (DTG).

We employed two different types of aqueous solutions, mimicking urine [20, 21], with our solvent (HA) introduced in several different concentrations. 

The physic-chemical formalism of the kinetics of dissolution of kidney stones has been developed, in details, in our paper [15]. One can see that simple formulae can be obtained, describing the effect of complex forming agents (present in the solution at various concentrations) on supersaturation, solubility, and growth velocity of CaOX crystals growing or dissolving in a solution, resembling human urine. It can be shown that if we introduce an increasing concentration *C*_H_ (e.g., HA) of a Ca^2+^-binding complex forming agent, having a solubility constant *K*_H_ into the solution, a linear dependence between the solubility *S*_H_ and *C*_H_ for Ca^2+^ ≫ C_2_O_4_^2−^ can be predicted by

(1) SH≈So·(1+KHCHα′),

where *α*′ is the *α*-factor in the absence of the complex forming agent H (here indicating hippuric acid). 

 Thus, the dependence of the supersaturation Δ*μ* on *C*_H_ for the physiologically significant case Ca^2+^ ≫ C_2_O_4_^2−^, determining the supersaturation in urine is

(2) Δμ≈Δμo−(12)·ln⁡(KHCHα′),

where Δ*μ*_o_ is the supersaturation in respect to the CaOX-precipitation without HA added.

 It is also of significant interest that in the case of the dissolution of CaOX-concrements in the presence of a fixed initial concentration of CaOX (or—which in the case of Ca^2+^ ≫ C_2_O_4_^2−^ is the same—in the presence of constant concentration *C*_o_* of oxalic anions Ca^2+^) we have to rewrite (1) as follows:

(3) SH≈So(1+KHCHα′)−Co∗.

Thus, a plot of *S*_H_ versus *C*_H_ should result in a straight line with a slope of −*S* · *K*_H_/*α*′ cutting from the ordinate axis a segment *S*_H_(0) = *S* − C_o_*. In this way, both *S*_H_ and *K*_H_ can be determined at a known value of *α* (according to data in Robertson et al. [22], *α*′ in human urine is approximately 2). 

Thus, depending on the concentration *C*_H_, that is, on the sign of Δ*μ* (i.e., Δ*μ* < 0 during growth, Δ*μ* < 0 during dissolution), growth or dissolution of CaOX concrements can be achieved simply by changing the concentration *C*_H_ of the hippuric acid added. 

## 3. Results

### 3.1. Clinical Laboratory Data

Table 1 shows reports by our laboratory on the urinary excretion of calcium, oxalate, creatinine, and others responsible for CaOX crystallization substances.

Mainly, urine concentrations of calcium and oxalate are responsible for calcium oxalate urine supersaturation, according to leading researchers [19, 22]. However, given the complexity of the pathogenesis of CaOX renal calculosis, the mean values of renal calculosis reagents (calcium [Ca^2+^] and oxalate [C_2_O_4_^2−^]) are not expected to be elevated in all analyzed cases. This is confirmed by our results. Due to the dispersion of the data, the average values of these indicators are within the accepted norms.

Only 10% of the patients have urinary calcium levels above 7.5 mMol/24 h (which is the upper limit of normal ranges [17]), but 39% of them have hyperoxaluria (oxalate above 0.45 mMol/24 h, according to [20]).

Table 2 shows the urine excretion (*μ*mol/24 h) of amino acids [23], mentioned above, in the groups of SF patients and controls. A mathematical estimation of the deviation from the observation over amino acids in patients has been made. It turned out that in the group of SFs, the investigated amino acid excretions were significantly decreased about 20% for serine and about 40% for Lysine. About 75% of the SFs showed also an excretion of HA lower than 5.6 mMol/24 h. 

### 3.2. Data of *In Vitro* Experiments

The solubility of CaOX in pure water at 25°C is about 4 mg/L (3,1·10^−5^ Mol/L) according to our results in [15, 16].

**Figure 1:**
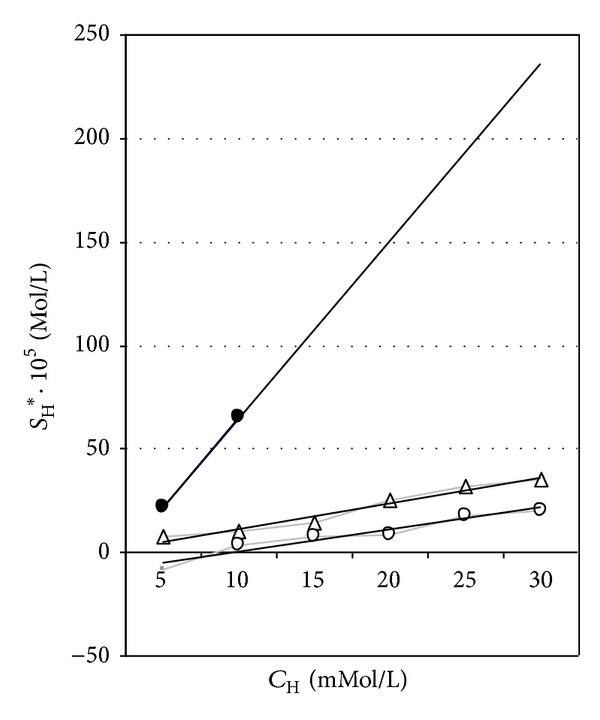
Solubility of calcium oxalate calculi in artificial urine as a function of hippuric acid concentration in three physiological solutions, according to the author's *in vitro* experiments. • solubility in zero saturation artificial urine (curve 1), △ solubility in artificial urine with lower saturation - 2,5 mmol/L Ca^2+^ and 0,02 mmol/L C_2_O_4_^2−^ ions (curve 2), and ∘ solubility in artificial urine with “normal” saturation as in “standard” human urine - 2,5 mmol/L Ca^2+^ and 0,2 mmol/L C_2_O_4_^2−^ ions (curve 3).

The solubility of calcium oxalate in artificial urine with zero supersaturation is considerably increased (8,7·10^−5^ mol/L) compared to its solubility, mentioned above, in pure water. This fact is due to the presence of complex ions ([Mg^2+^], citrate ions, etc.) in this solution, as predicted by their stability constant (*α*_*i*_), which is known from the analytical chemistry [24]. When HA is introduced into the same physiological solution, a dramatic change in solubility (up to 66·10^−5 ^mol/L) is observed, as shown in Figure 1. 

A similar effect of HA is also seen in artificial urine in which distinct supersaturation (due to the presence of normal concentration of [Ca^2+^] and medium concentration of [C_2_O_4_^2−^] ions) has been introduced (curves 2 and 3, Figure 1). In accordance to (1), a linear dependence of the solubility *C*_H_ is observed for each series of measurements, in which three different supersaturation values (zero, medium and normal) have been established. 

The effect of the increasing concentration of hippuric acid on the solubility of CaOX-concrements is clearly evident; the initially supersaturated solutions are transformed into undersaturated systems. The effect of the presence of [Ca^2+^] in the course of the straight lines 2 and 3 in Figure 1, as compared to the non-Ca^2+^ case (line 1 in the same figure) is to be noted. A sharp decrease in the slope of lines 2 and 3 is observed, too. Since the two solutions have the same [Ca^2+^] concentration, curves 2 and 3 are parallel, as expected from the formalism, as discussed above (see also [15]). 

## 4. Discussion

The majority of urinary calculi found in patients with urolithiasis are predominantly of calcium oxalate composition. Calcium and oxalate are the two urine substances responsible for CaOX crystallization. The results of our study indicate that CaOX SF patients are different from normal patients in regards to urinary oxalate excretion; about 39% of SFs excrete more oxalate than controls (*P* < 0.05) (see Table 1 and [20]). Only 10% of them have hypercalciuria. Few of them (4%), however, show hypercalciuria and hyperoxaluria together.

It seems natural to expect that the reduction of the total amount of oxalate that is present in the urine of SFs could give the key to prevention or to successful treatment of CaOX urolithiasis. It is known, that approximately 60% of urinary oxalate stones are derived from the endogenous metabolism of glycine, glycolate, and hydroxyproline, and 25% to 30% are the end product of dietary ascorbate metabolism. The remaining 10% to 15% come from dietary oxalate intake [8, 25, 26]. However, there is no means of decreasing endogenous oxalate production. Nevertheless, one may assume that decreasing exogenous oxalate intake may lessen urinary oxalate levels.

Detailed analysis performed in our previous study [15] indicates that HA is comparable, in its solubility effect, to the best known classical complex binders of [Ca^2+^] or [C_2_O_4_^2−^] ions in urine, that is, [Mg^2+^] and citrate anions. This can be seen from Table 3, in which the stability constant *K*_H_ of hippuric acid calculated according to our results (Figure 1 and (1)) is compared to *K*_*i*_-values of [Mg^2+^], Na-EDTA, and other known complex formers of CaOX. 

We have found that the mechanism of dissolution of CaOX calculi follows the Nernst model of a diffusion limited process as it is discussed in details in [15, 16]. 

At the same time, there is also a very a significant difference in the urinary HA concentration (*P* < 0.001) between both groups (see Table 1). Thus, it turns out that patients with oxalate stones generally excrete less HA than normal patients do. These results are meaningful from a physiological point of view considering the well-known fact that urinary HA excretion could be increased by oral administration of benzoic acid (BA) derivatives or salicylates. Moreover, the renal excretion of HA is not limited by the capacity of the renal tubular transport system, even at the highest excretion level obtained after administration of benzoates.

Sufficient data is available, confirming that the presence of HA in urine is a biological marker for the presence of organic aromatic compounds, for example, for benzoic or toluene exposure. Part of the toluene absorbed is eliminated by the exhaled breath, but a large percentage is oxidized in the organism to BA, conjugated with glycine, and excreted as HA in urine [12, 27, 28] according to the chemical reaction given below

(4) C6H5COOH+H2NCH2COOH=C6H5CONHCH2COOH+H2O
Benzoic  acid Glycine   Hippuric  acid  (HA)

**Figure 2:**
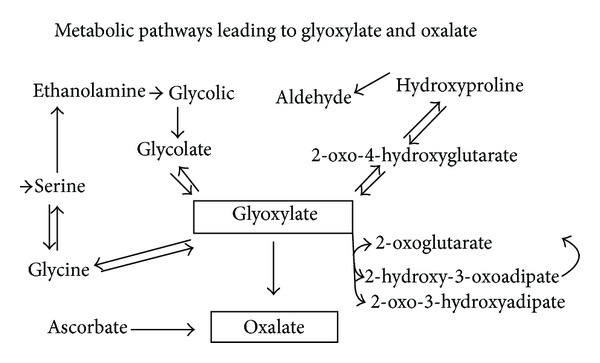
Metabolic pathway of oxalic acid.

Therefore, a high level of urinary HA may be *achieved* by the intake of BA. As a food preservative, BA (or sodium benzoate) is added to pickles, soft drinks, soya sauce, syrup, and caviar [28]. Besides, BA is naturally present in many fruits and vegetables, especially cranberries, prunes, and in coffee beans [29]. Sodium benzoate is also the constituent of several classical medicines (e.g., cough syrups). The origin of the increased presence of HA in human urine is presumably in many cases due to the BA present in the fruits mentioned above and also from the addition of benzoate preservatives to food. Along with this exogenous HA, an endogenous excretion should be considered too. It is known that HA may be bacteriostatic for Escherichia coli in concentration of 1 to 2 mg/mL [30, 31]. Last but not least, any of the paths mentioned above might exert an influence on the metabolic pathway of oxalic acid in the human organism (Figure 2).

 As benzoates bind glycine in the liver, it is expected that the glycine-glyoxylate-oxalate link can be influenced by moving the equilibrium to a decreased level of the endogenous oxalate.

 It is reasonable to assume that drug biotransformation interaction in the human organism, connected to the simultaneous conjugation of BA with glycine, could be a promising way for the development of future therapy methods for patients with CaOX calculi. We would like to add the possibility, we first mentioned in previous publications [15, 16], for the biological importance, which the multilateral role of HA output in the urine of patients with CaOX-lithiasis could have as a Ca complex-forming agent.

 In assessing the efficiency of HA as an eventual clinical solvent of CaOX calculi, one should also bear in mind that in the renal tract some conditions prevail, (continuous flushing of the calculus with fresh urine) under which the time necessary for the dissolution effect will be much shorter, as it is determined by the initial value of the dissolution rate. This possibility we confirmed in *in vitro* experiments with CaOX concrements, performed in confined volume and at physiological flow conditions, which correspond to a possible *in vivo *experiment in a human kidney pelvis (see [32]).

Not only does it even seem that HA is the natural regulator of CaOX supersaturation in human urine, but it also appears to be such a regulator in mammal urine, in general. This follows from other known experimental findings on the very low frequency of calcium oxalate stone formation in herbivorous mammals [15]. The concentration of HA in mammal urine is, as a rule, considerably higher than its level in human urine. A classical and well-studied case in this respect gives the urine of horses (from where Justus von Liebig derived the name of the hippuric acid, many years ago).

Taking into account the metabolism of benzoic acid and its salts, as described above, a good alternative for metaphylaxis of the CaOX precipitation of nutrients would be added to the sodium benzoate. According to the so-called hepatic sample of Quick [14], taking 4 gr of sodium benzoate with tea, leads to separation of 6 gr of hippuric acid. Sodium benzoate is found in many berries, some formulations, and so forth, so it is not difficult to raise the urinary excretion and to establish an appropriate diet or medication.

One should expect that by administering and applying benzoic acid, for a short period of time, a considerable higher concentration of HA in stone formers with CaOX calculi, corresponding to the physiological norm of this substance in urine, could be and is in fact achieved. Thus, a negative supersaturation in respect to CaOX-precipitation, and even the direct dissolution of CaOX calculi situated in the kidney pelvis, or elsewhere in the urinary tract, could be expected. One proof of this is achieved by our *in vitro* results of solubility of about 30 mMol/L stone.

The role and influence of drugs, containing benzoic acid derivatives which may provoke such effects, could be of great importance to a future medication. The amount of benzoates in a drug treatment will be the subject of future research. However, the important fact is that the salts of benzoic acid are contained in a number of berries and it may advantageously be applied as an appropriate diet for the prevention of CaOX nucleation.

## Figures and Tables

**Table 1:** Urinary excretion of inorganic components and some trace elements in patients with CaOX calculosis.

	Data, according [17]	Controls mMol/24 h	Stone-formers mMol/24 h	*P*
Calcium	2.5–7.5	3.9 ± 1.9	5.6 ± 2.1	*P* < 0.05
Oxalate	0.45	0.3 ± 0.17	0.46 ± 0.25	*P* < 0.001
Phosphorous	10.9–32.3	15.0 ± 10.4	19.2 ± 9.2	*P* < 0.05
Uric acid	1.19–4.16	1.9 ± 0.7	2.3 ± 1.5	*P* < 0.05
Creatinine	♂ 8.8–17.6 ♀ 5.3–13.2	10.4 ± 5.2	11.8 ± 6.1	*P* < 0.05
Magnesium	7.1–11.7	5.1 ± 1.9	3.9 ± 0.9	*P* < 0.05
Volume, mL	1000 ± 1500	1490 ± 350	1050 ± 300	*P* < 0.001
pH	6.0–6.1	6.0 ± 0.4	6.1 ± 0.3	NS

(*P*): statistically different from controls.

**Table 2:** Mean ± SEM values (*μ*mol/24 h) of urinary amino acids in controls and in the SFs.

Amino acids x.[*μ*mol/24 h]	Data, according [23]	Controls	Stone-formers
Serine (Ser)	584	392.3 ± 92.3	240.6 ± 202.6*
Glycine (Gly)	984	1064.7 ± 426.1	841.5 ± 439.9
Hippuric acid (HA), mMol/24	5.6–14 [17]	19.5 ± 8.4	1.9 ± 0.6*

*(*P* < 0.001) statistically different from controls.

**Table 3:** Hippuric acid as a solvent of calcium oxalate: comparison with classical complex-forming agents.

Ligand	Complex-former	Solution	*T* °C	pH	*K*_*i*_, L/mol	Reference
Na-EDTA	Ca^2+^	Physiological solution	25	7,0	5,0·10^5^	[15]
Mg^2+^	C_2_O_4_^2−^	0,3 M NaCl	37	—	5,6·10^3^	[19]
Mg^2+^	C_2_O_4_^2−^	0,3 M NaCl	25	5,0	4,0·10^3^	[15]
Citric anion	Ca^2+^	Pure water	25	—	5,0·10^2^	[22]
Hippuric acid	Ca^2+^	Zero saturated artificial urine	25	5,5	7,0·10^3^	[15]
